# Determination of Antimicrobial and Toxic Metal Removal Activities of Plant‐Based Synthesized (*Capsicum annuum* L. Leaves), Ecofriendly, Gold Nanomaterials

**DOI:** 10.1002/gch2.201900104

**Published:** 2020-02-20

**Authors:** Mehmet Firat Baran, Hilal Acay, Cumali Keskin

**Affiliations:** ^1^ Medical Laboratory Techniques Vocational Higher School of Healthcare Studies Mardin Artuklu University 47200 Mardin Turkey; ^2^ Health Science Faculty Department of Nutrition and Dietetics Mardin Artuklu University 47200 Mardin Turkey

**Keywords:** antimicrobial activity, nanoparticles, removal activity, SEM, toxic metals, UV–vis, XRD

## Abstract

Nanoparticles are valuable materials with widespread use. The fact that these materials are obtained by biological resources with an environmentally friendly method contributes to the development of studies in this field. Gold nanoparticles (AuNPs) from waste vegetable sources (green leaves of *Capsicum annum* L.) are economically and easily synthesized. The obtained particles are characterized by UV–vis spectroscopy (UV–vis), Fourier transform infrared spectroscopy (FTIR), X‐ray diffraction (XRD), and scanning electron microscopy (SEM) analysis. The antimicrobial activity of the particles on the pathogenic microorganisms *Escherichia coli* ATCC 25922, *Staphylococcus aureus* ATCC 29213, *Bacillus subtilis* bacteria, and *Candida albicans* yeast are found to have a significant suppressive effect. The removal activities of eight toxic metals (Pd, Cd, Fe, Ni, Co, Mn, Zn, Pb) in Diyarbakır drinking water and artificially prepared water within different pHs are investigated. Gold nanoparticles synthesized from *Capsicum annuum* L. leaves are found to be effective in toxic metal removal in water samples.

## Graphical abstract

Plant‐based synthesis of nanoparticles is environmentally friendly; the application stages are easier and cheaper, making it more advantageous than other chemical methods. Gold nanoparticles are widely used in medicine, catalysis, biosensors, and optical‐electronic devices. In addition, gold and silver nanoparticles have been used extensively in the pollution (water, paint) removal of artificial and natural waters in recent years.

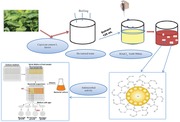

## 1 Introduction

**Figure 1:**
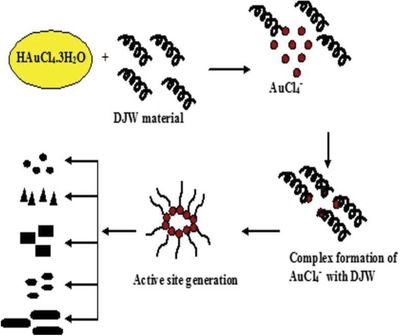
Schematic representation of the synthesis of gold nanoparticles from waste vegetable source.^[^ 8 ^]^

Nanotechnology is an area that includes nanoparticles that can be obtained biologically, physically, and chemically and their functions. Nanoparticles have improved surface area width, strength, and conductivity, making them suitable for use in various fields. Metallic particles such as gold, silver, platinum, and palladium are the most well known of these particles.^[^ 1, 2 ^]^ Gold nanoparticles are important nanomaterials and they are widely used in catalysis reactions, biosensor applications, optical‐electronic devices, and some medicinal treatments.^[^ 3 ^]^ The resistance of microorganisms to unconsciously used antibiotics causes serious problems in the fight against pathogenic microorganisms. Therefore the search for alternative antimicrobial agents is very important.^[^ 4 ^]^ As an antimicrobial agent, AuNPs synthesis with waste biological sources is quite valuable for biocompatibility.^[^ 5 ^]^ Gold nanoparticles are can be synthesized chemically and physically, but these methods are very precious, and many toxic chemicals are used in the synthesizing and not being environmentally friendly is of great concern. It is also important to use unused biological resources to reduce costs. The synthesis with waste biological sources against these methods is environmentally friendly; the application stages are easier and cheaper, making it more advantageous than other methods.^[^ 6 ^]^ Biomolecules found in the biological sources used by this method allow the reduction of gold (III) chloride trihydrate (HAuCl_4._3H_2_O) and the formation of AuNPs.^[^ 3, 7, 8 ^]^ Schematic representation of the synthesis of gold nanoparticles from a waste vegetable source is shown in **Figure** **1**.

On the other hand, one of the most important problems in nature is environmental pollution. The main cause of pollution is the contamination of the ecosystem with industrial processes. So, it is necessary to find new methods or alternative materials for removing toxic metal ions from the ecosystem. Ecofriendly nanoparticles, synthesized naturally, have been used extensively in recent years in the studies of pollution (water, paint) removal. Especially gold and silver nanoparticles have been used extensively in the pollution removal of artificial and natural waters in recent years.^[^ 9 ^]^ This research deals with the evaluation of the antimicrobial activities of gold nanoparticles synthesized from plant (*C. annuum* green leaves) extracts and the determination of toxic metals removal activities of greenway synthesized gold nanoparticles from waste and artificial waters samples.

## 2 Results and Discussion

### 2.1 UV–Vis Spectrophotometer Analysis

**Figure 2:**
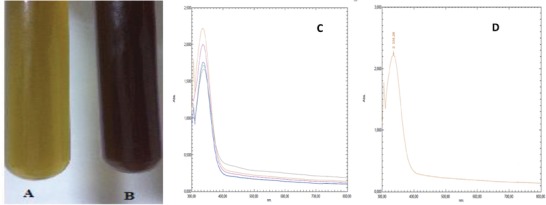
UV–vis showing the formation and presence of AuNPs. Analysis results data. A) Before synthesis, B) after synthesis, C) absorbance data obtained from periodic measurements, D) maximum absorbance data.

After the plant extract and gold (III) chloride trihydrate (HAuCl_4._3H_2_O) were mixed, the color change from yellow to dark red was observed. The maximum wavelength was measured on the UV–vis spectrophotometer (335.26 nm) depending on the color change (**Figure** **2**).

In a study conducted with the ecofriendly synthesis of *Gloriosa superba* leaf extract, AuNPs have shown maximum absorbance at 538 nm.^[^ 8 ^]^ Another study reported that AuNPs showed strong peaks at 300–550 nm (*Marsdenia tenacissima* aqueous leaf extract).^[^ 10 ^]^ *Coleus aromaticus* leaf extracts in the synthesis study of 544 nm peaks were interpreted by the presence of AuNPs.^[^ 5 ^]^

### 2.2 Fourier Transform Infrared Spectroscopy Measurement

**Figure 3:**
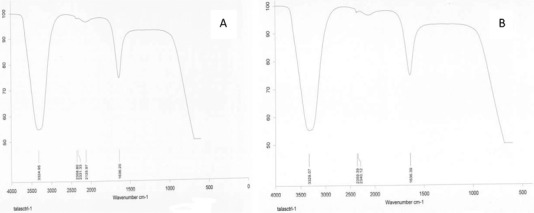
FTIR analysis result data. A) Extract, B) evaluation of functional groups after synthesis.

Functional groups involved in the reduction of AuNPs were examined by FTIR analysis. The shifts at 3324–3329, 2100 and 1636 cm^−1^ show that the —OH, —CN and C=O groups may be involved in the reduction, respectively (**Figure** **3**). In the environmentally friendly studies for the synthesis of AuNPs, these functional groups were reported to be functional groups responsible for the reduction. In the synthesis study with plant‐derived *Terminalia arjuna* extract, the —CN group was found to be among those involved in the reduction.^[^ 11 ^]^ In a study conducted with *Cystoseira baccata* extract, indicated that the —OH group may be responsible for the reduction.^[^ 12 ^]^ In another study, it is mentioned that these functional groups are involved in reduction.^[^ 6 ^]^

### 2.3 X‐Ray Diffraction Analysis

**Figure 4:**
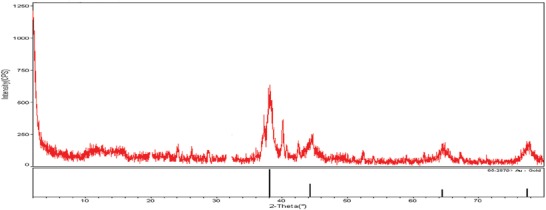
Investigation of crystal structure and gold phases of AuNPs by XRD analysis.

Crystal structures of AuNPs were analyzed by XRD. The results show that the sharp peaks at 111°, 200°, 220°, and 311°, corresponding to 2θ, are the peaks that show the crystal structure of AuNPs (**Figure** **4**).

The numerical values ​​of these peaks were found to be 38.32, 44.81, 64.45, and 77.68. In their synthesis study with *Osmanthus fragrans* leaf extract, they stated that the peaks at 111°, 200°, 220°, and 311° show the crystal structure of AuNPs.^[^ 13 ^]^ *Croton caudatus* Geisel leaf extracts in the study (111), (200), (220), (311) peaks and 38.1, 44.0, 64.4, and 74.4 values ​​of these peaks were reported to belong to the crystal structure of AuNP.^[^ 14 ^]^ In other environmentally friendly synthesis studies, it was observed that AuNPs showed this character in XRD data.^[^ 15, 16, 17 ^]^ Based on this information, the crystal size of AuNPs was calculated as 13.71 nm using the Debye‐Scherrer formula. In the synthesis study with *Annona squamosa L*. fruit extract, the crystal particle size of AuNPs was calculated as 4.6 nm using this formula.^[^ 18 ^]^ In the other two biological synthesis studies, the same formula was used and the crystal particle size was calculated as 23.7 and 18 nm, respectively.^[^ 5, 11, 19 ^]^

### 2.4 Scanning Electron Microscope and Energy‐Dispersive X‐Ray Spectroscopy

**Figure 5:**
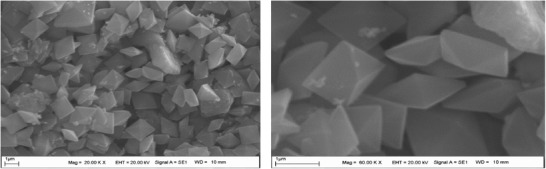
Evaluation of the morphology of AuNPs in SEM results.

The SEM images were examined, it was determined that AuNPs showed morphological character with the triangular pyramid, square and rectangular shape (**Figure** **5**). Researchers reported that the gold nanoparticles obtained in the synthesis with *Sphaeranthus indicus* extract were spherical in appearance.^[^ 20 ^]^

**Figure 6:**
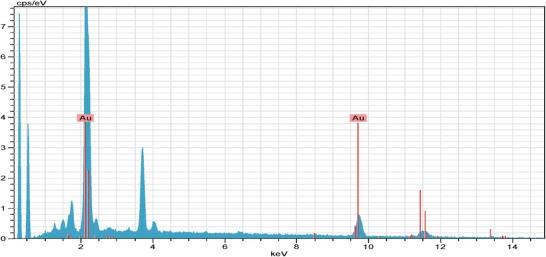
Investigation of the elemental composition of AuNPs by EDX analysis.

In another study using various biological sources, researchers mentioned that AuNPs are square, rectangular, triangular, and pyramidal in different shapes.^[^ 1 ^]^ In another study, researchers reported that AuNPs have shown a triangular appearance.^[^ 21 ^]^ Similar studies support the data obtained from the images are available.^[^ 22, 23 ^]^ The EDX data are analyzed, it is seen that sharp gold peaks are formed clearly. In addition, when the EDX pattern is examined, it is seen that the majority of the nanoparticles are in elemental form (**Figure** **6**). EDX data obtained from other gold nanoparticle studies are in line with our findings.^[^ 3, 24, 25 ^]^

### 2.5 Investigation of Antimicrobial Activity

AuNPs have a suppressing effect on the growth of microorganisms. By increasing the amount of ROS, it disrupts the cell wall and membrane structure, causing damage to vital functions such as DNA, RNA synthesis, and affinity to ROS.^[^ 5, 26 ^]^ The antimicrobial activity of AuNPs synthesized with plant (*C. annuum* green leaves) extract was investigated. It was determined that these particles have a strong antimicrobial effect at low concentrations. Compared with 5 × 10^−3^ m gold chloride solution and antibiotics, we found that lower concentrations were effective. AuNPs inhibited the growth of *S. aureus*, *B. subtilis*, *E coli*, and *C. albicans* (yeast) at concentrations of 0.112, 0.055, 0.224, and 0.028 mg mL^−1^ respectively (**Table** **1**).

**Table 1:** MIC values of synthesized AuNPs on gold(III) chloride trihydrate solution and vancomycin, fluconazole, colistin antibiotics on *S.aures*, *B. subtilis C. albicans*, and *E. coli* microorganisms

Organism	AuNPs nanoparticles [mg mL^−1^]	HAuCl_4._3H_2_O solution [mg mL^−1^]	Antibiotic [mg mL^−1^]
*S. aureus* *ATCC 29213*	0.112	0.5	1
*B. subtilis*	0.055	0.10	1
*E. coli* *ATCC25922*	0.224	0.25	2
*C. albicans*	0.028	0.25	2

It was said that the AuNPs obtained in the synthesis with *Gelidium amansii* by environment friendly method showed activity above 100 mg mL^−1^ concentration.^[^ 27 ^]^ In a similar study, AuNPs synthesized by extracting vegetables from the waste in the market were investigated by *Staphylococcus sp*. concentrations of 25–100 mg mL^−1^ were said to be effective.^[^ 28 ^]^ In another study with *Anacardium occidentale*, it was reported that 20–40 mg mL^−1^ concentrations were effective on *E. coli* and *B. subtilis*.^[^ 26 ^]^

### 2.6 Determination of Toxic Metals Removal Activity

**Figure 7:**
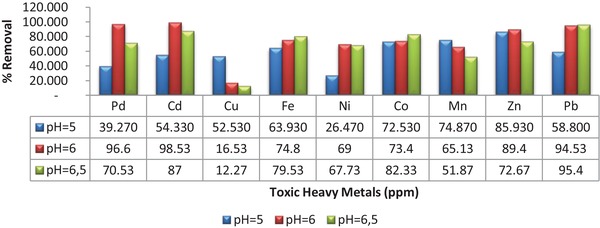
Toxic metals % removal activities of gold nanoparticle adsorbents at different pH.

Synthesized gold nanoparticles were used as an adsorbent to remove toxic metals in waters. Toxic metals were effectively removed from artificially prepared solutions (pH 5, 6, 6.5) with the synthesized nanoparticles (**Figure** **7**).

The best removal activity for Pd (0.051), Cd (0.022), Ni (0.465), Zn (0.159), and Pb (0.082) toxic metals was determined at pH 6. Mn and Fe showed the best removal activity at pH 5 and pH 6, respectively.^[^ 29 ^]^ It was determined that the gold nanoparticle adsorbent could be used as an alternative biological remover in artificially prepared aqueous solutions (**Table** **2**).

**Table 2:** Toxic heavy metal removal of gold nanoparticles at different pHs in an artificially prepared wastewater environment (Co = 1.5 ppm, speed: 240 rpm, *m* = 0.010 g, time = 120 min, temperature = 25 °C, *V* = 100 mL)

Toxic elements	pH 5	pH 6	pH 6.5
Pd	0.911 ± 0.043	0.051 ± 0.001	0.442 ± 0.011
Cd	0.685 ± 0.003	0.022 ± 0.005	0.195 ± 0.018
Cu	0.712 ± 0.013	1.252 ± 0.004	1.316 ± 0.001
Fe	0.541 ± 0.030	0.378 ± 0.015	0.307 ± 0.001
Ni	1.103 ± 0.070	0.465 ± 0.004	0.484 ± 0.002
Co	0.412 ± 0.061	0.399 ± 0.003	0.265 ± 0.003
Mn	0.377 ± 0.001	0.523 ± 0.006	0.722 ± 0.002
Zn	0.211 ± 0.041	0.159 ± 0.005	0.410 ± 0.005
Pb	0.618 ± 0.003	0.082 ± 0.002	0.069 ± 0.005

Optimized conditions were applied to Diyarbakır drinking water (pH 6) samples. The initial and equilibrium concentrations of the toxic metals in the test sample were measured and the % removal activities were calculated. The highest removal activity was measured in Pd (75.76%) and the lowest activity was observed in Mn (21.62%) (**Table** **3**).

**Table 3:** Toxic heavy metal removal of gold nanoparticle as an adsorbent in a water sample taken from Diyarbakır drinking water network (Speed: 240 rpm, *m* = 0.010 g, time = 120 min, temperature = 25 °C, *V* = 100 mL)

Toxic elements	*C*_O_ a)	*C*_E_ a)	Removal%
Pd	0.52 ± 0.003	0.19 ± 0.001	63.46
Cd	0.98 ± 0.001	0.39 ± 0.005	60.20
Cu	15.84 ± 1.6	7.68 ± 0.004	51.50
Fe	8.30 ± 0.022	2.64 ± 0.015	68.20
Ni	15.08 ± 0.77	8.72 ± 0.004	42.18
Co	0.98 ± 0.002	0.75 ± 0.003	23.47
Mn	0.74 ± 0.005	0.58 ± 0.006	21.62
Zn	106.3 ± 0.023	68.70 ± 0.005	35.37
Pb	0.66 ± 0.078	0.16 ± 0.002	75.76

a) *C*_0_, initial concentration; *C*_E_, equilibrium concentration.

## 3 Conclusions

The fact that nanomaterials are used in many different fields in today's world makes the obtaining of these materials valuable. The synthesis of biological sources is environmentally friendly, cheap, and easy to make this method attractive. The development of resistance of pathogenic microorganisms has led to an increase in the demand for different antimicrobial agents. AuNPs synthesized from waste green leaves of a pepper plant in a simple, economical, and environmentally friendly way. The synthesized gold nanoparticles were showed strong antimicrobial activity with low concentrations. Additionally, the obtained gold nanoparticles can be used as an effective adsorbent in water pollution removal. The synthesis of AuNPs can be developed and used for medical (antimicrobial and anticancer agents) and in different industries applications.

## 4 Experimental Section

##### Preparation of Plant (*Capsicum annuum* L. Green Leaves) Extract and Gold (III) Chloride Trihydrate (HAuCl_4_.3H_2_O)

**Figure 8:**
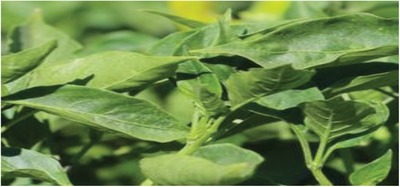
Plant material (*Capsicum annuum* L.) used for extract preparation.

The green leaves of the hot pepper (*Capsicum annuum* L.) grown in the Diyarbakır region were collected in the Hevsel garden of Sur district near the Tigris River after the harvest (**Figure** **8**).

It was washed with tap water followed by distilled water and dried under room conditions. The dried plant was reduced in size. Ten grams were taken and boiled with 250 mL distilled water. The prepared extract was stored at +4 °C to be used for synthesis after a series of filtration. Tetrachloroauric(III) (Sigma Aldrich) acid was commercially purchased. 5 × 10^−3^ m aqueous solution of gold (III) chloride trihydrate (HAuCl_4._3H_2_O) was prepared. 0.1 molar nitric acid solution was used for adsorbent recovery. The gold adsorbent was washed with the prepared acid solution and then dried. The recovery rate was calculated as 85%.

##### Mix‐Metal Ion Adsorption at Low Concentrations

The required dilutions were made for each (1000 ppm) stock solution of Pd(II), Co(II), Mn(II), Ni (II), Cd(II), Pb(II), Cu(II), Fe(II), and Zn(II) and metal ion solutions with an initial concentration of 1.5 ppm were prepared. 0.010 g of ecofriendly gold nanoparticles adsorbents was weighed and 50 mL of metal solutions were added into 100 mL flask. The prepared solution was shaken at 25 °C to equilibrate the reaction. Samples from the reaction flask were centrifuged before being instrumental analysis (ICP‐OES). To measure the accuracy of the ICP‐OES, the commercially purchased CRM wastewater sample (EPA) was used (**Table** **4**).

**Table 4:** CRM value of certified wastewater samples

Toxic elements	Certified waste water values [µg L^−1^]	Reading value [µg L^−1^]	Quality control [µg L^−1^]	Profession testing [µg L^−1^]
Cu	744 ± 0.001	691 ± 0.006	673–811	632–856
Cd	184 ± 0.050	196 ± 0.018	163–195	156–212
Pb	631 ± 0.046	603 ± 0.062	569–694	536–726
Ni	1.590 ± 0.045	1.563 ± 0.061	1260–1520	1230–1560
Zn	1.520 ± 0.046	1.440 ± 0.0076	1380–1670	1290–1750
Fe	2.750 ± 0.121	2.840 ± 0.038	2480–3050	2340–3160

The absorbed metal equilibrium concentrations (*C*_E_) were subtracted from the initial metal concentrations (*C*_0_). The resulting difference was divided into initial metal concentration (*C*_0_). The % removal was calculated by multiplying the obtained value by 100.^[^ 9 ^]^

(1) $$\%A\;=\frac{\left(C_{0}-C_{E}\right)}{C_{0}}\;\times \;100$$

##### Multiple Metal Adsorptions in Drinking Water

The adsorption capacity of the adsorbent used in the aqueous solutions of toxic metals was determined by the batch method. Gold nanoparticle adsorbent was applied to Diyarbakır drinking water.

##### Synthesis and Characterization of Gold Nanoparticles

125 mL of extract and 500 mL of 5 × 10^−3^ m gold (III) chloride trihydrate (HAuCl_4._3H_2_O) solution were combined in 1000 mL followed by constant bending at room conditions after simple shaking. The color change was observed. The color conversion from yellow to dark red was measured with Perkin Elmer one UV–Vis spectrophotometer depending on the time. Absorbance data were evaluated. To evaluate the phytochemicals and functional groups responsible for synthesis (responsible for reduction), FTIR analysis was performed with Perkin Elmer Spectrum One instrument. After synthesis, the liquid fraction was centrifuged at 6.000 rpm for 15 min with OHAUS FC 5706 model centrifuge. The precipitate was washed with a series of distilled water. It was dried in an oven at 70 °C. The element composition of the particles was evaluated by RadB‐DMAX II computer‐controlled X‐ray diffractometer (EDX). The morphology of the particles was examined by scanning electron microscope EVO 40 LEQ (SEM) device images RadB‐DMAX II computer‐controlled X‐ray diffractometer XRD device analysis results were used to evaluate the crystal structure of the particles. The crystal particle sizes of the particles were calculated using the device data and the Debye‐Scherrer equation.

(2) $$D=K\lambda \text{/}\left(\beta \;\text{cos}\theta \right)$$

In equation 2, *D* is the crystal diameter (nm) of the particle; *K* is the constant number (0.90); λ is the wavelength X‐ray (1.5406 Å); half is the maximum peak width at half height (rad.); Bra specifies the Bragg angle (degrees).^[^ 30 ^]^

Inductively coupled plasma optical emission spectrometry (ICP‐OES, Perkin‐Elmer Optima 5300) was used for the determination of toxic metals removal in waters samples.

##### Investigation of Antimicrobial Activities of Gold Nanoparticles

The antimicrobial effect of AuNPs was investigated by the microdilution method to determine minimum inhibitory concentration (MIC). Gram‐negative Gr (‐) *Escherichia coli* ATCC 25922, Gram‐positive Gr (+) *Staphylococcus aureus* ATCC 29213, *Bacillus subtilis* bacteria, and *Candida albicans* yeast were used. Mueller Hinton medium was placed in microplate wells and a series of dilutions were performed by adding the solution containing AuNPs obtained by synthesis. The concentration of microorganisms in the mixture was 0.5 according to Mcfarland standard^[^ 31, 32, 33 ^]^ and the mixture was incubated overnight at 37 °C. For control and comparison, minimum inhibition concentration (MIC) values were examined using colistin for Gram‐negative, vancomycin for Gram‐positive and fluconazole antibiotics for *C. albicans* and 5 × 10^−3^ m HAuCl_4_.3H_2_O aqueous solution.

## Conflict of Interest

The authors declare no conflict of interest.
